# Persistent Organic Pollutants and Early Menopause in U.S. Women

**DOI:** 10.1371/journal.pone.0116057

**Published:** 2015-01-28

**Authors:** Natalia M. Grindler, Jenifer E. Allsworth, George A. Macones, Kurunthachalam Kannan, Kimberly A. Roehl, Amber R. Cooper

**Affiliations:** 1 Division of Reproductive Endocrinology and Infertility, Department of Obstetrics and Gynecology, University of Colorado Denver, Aurora, Colorado, United States of America; 2 Division of Reproductive Endocrinology and Infertility, Department of Obstetrics and Gynecology, Washington University in St. Louis, Barnes-Jewish Hospital, St. Louis, Missouri, United States of America; 3 Department of Biomedical and Health Informatics, University of Missouri—Kansas City School of Medicine, Kansas City, Missouri, United States of America; 4 Division of Maternal Fetal Medicine, Department of Obstetrics and Gynecology, Washington University in St. Louis, Barnes-Jewish Hospital, St. Louis, Missouri, United States of America; 5 Wadsworth Center, New York State Department of Health and Department of Environmental Health Sciences, State University of New York at Albany, Albany, New York, United States of America; University of Missouri, UNITED STATES

## Abstract

**Objective:**

Endocrine-disrupting chemicals (EDCs) adversely affect human health. Our objective was to determine the association of EDC exposure with earlier age of menopause.

**Methods:**

Cross-sectional survey using National Health and Nutrition Examination Survey (NHANES) data from 1999 to 2008 (n = 31,575 females). Eligible participants included: menopausal women >30 years of age; not currently pregnant, breastfeeding, using hormonal contraception; no history of bilateral oophorectomy or hysterectomy. Exposures, defined by serum lipid and urine creatinine-adjusted measures of EDCs, data were analyzed: > 90^th^ percentile of the EDC distribution among all women, log-transformed EDC level, and decile of EDC level. Multi linear regression models considered complex survey design characteristics and adjusted for age, race/ethnicity, smoking, body mass index. EDCs were stratified into long (>1 year), short, and unknown half-lives; principle analyses were performed on those with long half-lives as well as phthalates, known reproductive toxicants. Secondary analysis determined whether the odds of being menopausal increased with EDC exposure among women aged 45–55 years.

**Findings:**

This analysis examined 111 EDCs and focused on known reproductive toxicants or chemicals with half-lives >1 year. Women with high levels of β-hexachlorocyclohexane, mirex, p,p’-DDE, 1,2,3,4,6,7,8-heptachlorodibenzofuran, mono-(2-ethyl-5-hydroxyhexyl) and mono-(2-ethyl-5-oxohexyl) phthalate, polychlorinated biphenyl congeners −70, −99, −105, −118, −138, −153, −156, −170, and −183 had mean ages of menopause 1.9 to 3.8 years earlier than women with lower levels of these chemicals. EDC-exposed women were up to 6 times more likely to be menopausal than non-exposed women.

**Conclusions:**

This study of a representative sample of US women documents an association between EDCs and earlier age at menopause. We identified 15 EDCs that warrant closer evaluation because of their persistence and potential detrimental effects on ovarian function. Earlier menopause can alter the quantity and quality of a woman’s life and has profound implications for fertility, human reproduction, and our global society.

## Introduction

Industrialization has led to the production of thousands of chemicals used in the manufacturing of pharmaceuticals, personal care products, and other common household items. Although the persistence and bioaccumulation of certain chemicals have led to concerns about health consequences, data on potential toxic effects remain scarce. Understanding the health effects of common chemical exposures is challenging as exposures may vary by geographic, demographic, and socioeconomic factors, be expensive to measure, be correlated with other chemicals, and result in modest changes in health risk.

Endocrine-disrupting chemicals (EDCs), chemicals or combinations of chemicals that interfere with any aspect of *in vivo* hormonal action, are of particular concern.[1,2] EDC exposure has been linked to increased incidence of certain cancers, metabolic syndrome [3] and cardiovascular disease.[1,2] Moreover, exposure to these compounds has been linked to reduced sperm quality, earlier age of puberty, declines in fecundity, and increased rates of pregnancy complications.[1,4] However, whether there is a relationship between EDC exposure and age at menopause has not been explored on a large scale. If EDCs are associated with earlier menopause, the impact on a woman’s quality of life (e.g., vasomotor symptoms, mood, and memory changes) and longevity (e.g., osteoporosis and cardiovascular disease) may be profound.[5] Among several classes of EDCs, perfluorochemicals have been associated with early menopause.[6–8]

**Figure 1:**
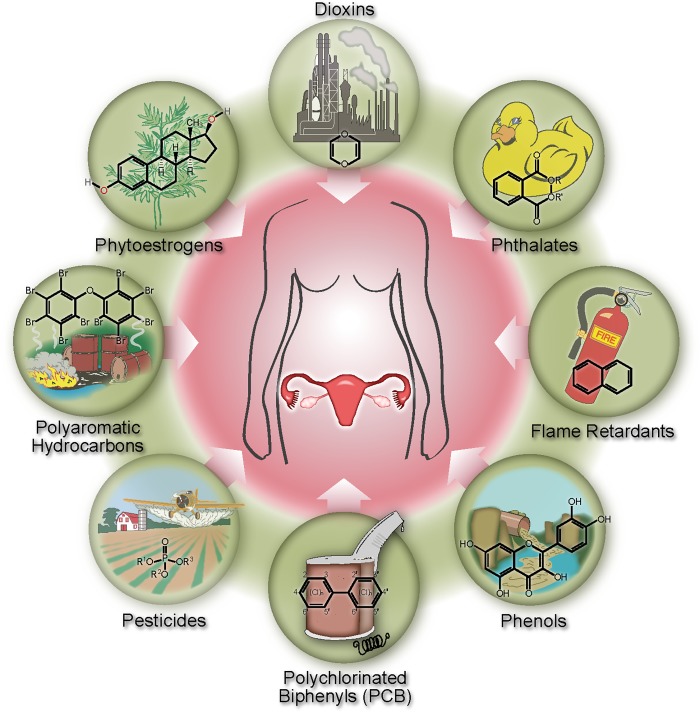
Evaluating groups of endocrine disrupting chemicals and their potential impact on female reproduction.

We sought to determine whether exposure to persistent EDCs such as PCBs and DDT was associated with earlier menopause. To do so, we examined data collected by the National Health and Nutrition Examination Survey (NHANES).[9] NHANES, administered by the Centers for Disease Control and Prevention (CDC), is a cross-sectional, nationally representative survey of the health and nutritional status of the US population. NHANES uses a complex, multistage probability design to obtain a representative sample of the civilian, non-institutionalized population. Specifically, we evaluated the serum or urine levels of 111 individual, potential EDCs from the following categories: dioxins (combustion byproducts), phytoestrogens (plant-derived estrogens), phthalates (plasticizers), polychlorinated biphenyls (PCBs, coolants), phenolic derivatives (phenols, industrial pollutants), organophosphate pesticides, surfactants, and polycyclic aromatic hydrocarbons (PAHs, combustion products) (Fig. 1).

## Methods

### Ethics statement

NHANES is a publicly available data set and all participants in NHANES provide written informed consent, consistent with approval by the National Center for Health Statistics Institutional Review Board.

### Study cohort

We examined data from all women in the 1999–2000, 2001–2002, 2003–2004, 2005–2006, and 2007–2008 survey periods (N = 31,575). At the time of this analysis, the entirety of the 2009–2010, 2011–2012, and 2013–2014 datasets were not publically available. We included women in the primary analysis if they were over 30 years of age (N = 13,705), menopausal (N = 2,159), and had been selected for EDC testing (N = 1,442). During each two-year survey period, 5000 individuals were chosen for participation in the study, and EDCs were measured in the serum or urine of random subsets. Our primary outcome of interest was age at last menstrual period in menopausal women. Exclusion criteria included: current pregnancy, breastfeeding, use of hormonal contraception or hormonal therapy, or history of hysterectomy or bilateral oophorectomy. Given the NHANES sampling techniques and weighting for study design characteristics,[9] our sample of 1,442 menopausal women who had a laboratory assessment of EDC levels represents a population of approximately 8,872,966 menopausal women across the US (Table 1).

**Table 1:** Demographic and reproductive characteristics and general health of eligible, menopausal NHANES participants and the subset of menopausal participants with EDCs measured.

	**All women 30+ years**	**All menopausal women**	**Menopausal women with any EDC measured**	**Women 45–55 years with any EDC measured**
N	13,705	2159	1442	2234
US Population size represented (weighted N)	***87,287,033***	***13,281,193***	***8,872,966***	***18,939,894***
*Sociodemographic characteristics*				
Age, mean (SE)	52.1 (0.2)	60.7 (0.4)	60.9 (0.5)	49.8 (0.1)
Race/ethnicity				
White, non-Hispanic	72.0%	76.0%	75.7%	70.6%
Black, non-Hispanic	11.6%	9.2%	9.1%	12.6%
Mexican American	6.1%	3.6%	3.4%	5.7%
Other Hispanic	4.9%	7.1%	7.3%	4.5%
Other race	5.5%	4.1%	4.5%	6.6%
Education				
Less than high school	7.1%	9.9%	9.1%	5.2%
High school or equivalent	38.1%	44.1%	43.8%	23.3%
Some college or college graduate	54.6%	45.9%	47.1%	59.8%
Born in the United States	85.8%	86.9%	85.6%	86.3%
Family poverty level				
Below the poverty level (<100%)	12.1%	13.0%	12.5%	9.8%
At the poverty level (100–199%)	19.4%	20.5%	20.9%	14.4%
Above the poverty level (≥200%)	68.5%	66.5%	66.6%	75.8%
*Reproductive characteristics*				
Age at menarche, mean (SE)	12.8 (0.0)	12.8 (0.1)	12.8 (0.1)	12.7 (0.0)
Age at menopause, mean (SE)	—	45.5 (0.4)	45.4 (0.4)	44.6 (—)
Number of pregnancies, mean (SE)	3.4 (0.0)	3.9 (0.1)	3.8 (0.1)	3.1 (0.1)
Number of live births, mean (SE)	1.0 (0.0)	2.8 (0.1)	2.8 (0.1)	0.9 (0.0)
Number of children breastfed at least 1 month, mean (SE)	2.2 (0.0)	2.4 (0.1)	2.4 (0.1)	2.0 (—)
*General health and behaviors*				
Body mass index, mean (SE)	28.7 (0.2)	28.8 (0.2)	28.7 (0.2)	29.1 (0.2)
Number of alcoholic drinks/day, mean (SD)	1.8 (0.1)	1.8 (0.1)	1.8 (0.1)	2.0 (—)
Smoking history				
Never smoked 100 cigarettes	57.4%	55.2%	54.7%	53.4%
Current smoker	19.5%	18.4%	20.2%	24.2%
Past smoker	23.1%	26.4%	25.1%	22.4%

### Chemicals assayed

We examined the data reported for individual chemicals within eight categories of EDCs: dioxins/furans, PCBs, phthalates, phytoestrogens, phenols, pesticides, PAHs, and surfactants (Fig. 1). Chemical analytes were measured by the National Center for Environmental Health Laboratories (CDC, Atlanta, GA) by gas chromatography-mass spectrometry or by high-performance liquid chromatography-mass spectrometry. Of the 176 chemicals analyzed, lipid- and creatinine-adjusted levels for 111 individual chemicals were included in this study (S1 Table). Sixty-seven EDCs were excluded because all results were below the limit of detection or there were no menopausal women with observed values in the top decile of exposure; PCB-28 and op DDT were similarly excluded from analysis given the rare detection of these chemicals in humans (S2 Table).

Given the limitations inherent in the use of cross-sectional data, wherein exposure measurements occur at variable times after the outcome of interest, our primary analysis focused on EDCs with half-lives of more than one year; although phthalates have biological half-life less than 24 hours in humans, we included them in our analysis because of these chemicals’ well documented effects on reproduction.[10–12] Determining the biological half-life of EDCs is challenging as each chemical is affected by unique mechanisms of decay, metabolism, individual body composition, and genetic predisposition. Therefore, we used only available literature from publications of the Agency for Toxic Substances & Disease Registry (CDC). We grouped chemicals as having biological half-lives of more than one year (n = 28), less than one year (n = 34), or not available (n = 49) (Tables 2 and S1). We included phthalates in our analyses due to concern regarding high and often continued levels of human exposure [13] as well as their known detrimental impact on reproductive outcomes, despite suspected short half-lives [2,14,15]. Due to the limitation of half-life, metabolism, and tissue specific data in humans, results for the chemicals with short or unknown half-lives are also included in supplemental information provided (S1 and S2 Tables).

**Table 2:** Distribution of pervasive EDCs associated with earlier menopause among women sampled by NHANES, 1999–2008, based on threshold analysis.

**EDC**	**Compound Name**	**Source**	**Min (ng/g)**	**Max (ng/g)**	**Median (ng/g)**	**90th percentile (ng/g)**
PCB	PCB-74	Serum	0.66	180	6.75	24.6
	PCB-99	Serum	0.65	304	4.95	16.3
	PCB-105	Serum	0.07	76.8	3.61	6.66
	PCB-118	Serum	0.90	354	7.50	32.8
	PCB-138	Serum	1.38	432	18.2	63.6
	PCB-153	Serum	1.34	524	26.3	84.4
	PCB-156	Serum	0.04	54.1	4.68	13.2
	PCB-170	Serum	0.06	115	7.70	24.0
	PCB-183	Serum	0.04	48.3	3.69	6.54
Pesticide	p,p’-DDE	Serum	1.56	27900	243	1430
	Beta-hexachlorocyclohexane	Serum	1.06	3500	8.24	49.5
	Mirex	Serum	0.50	2960	3.89	9.46
Dioxin/furan	1,2,3,4,6,7,8-heptachlorodibenzofuran	Serum	0.80	367	7.22	15.7
Phthalate	Mono-(2-ethyl-5-hydroxyhexyl)phthalate	Urine	0.14	7438	17.1	95.2
	Mono-(2-ethyl-5-oxohexyl)phthalate	Urine	0.14	4743	11.4	59.5

### Primary analysis

The primary outcome of interest was age at last menstrual period among menopausal women. Women who had not reported a period in the last 12 months were asked, “About how old were you when you had your last menstrual period?” Previous work has demonstrated that recall of age at menarche and menstrual cycle regularity is accurate in surveys.[16]

**Threshold analysis**. To determine whether the highest observed levels of EDCs were associated with age of menopause, exposure was defined as values in the top decile of the distribution among all women assessed for that EDC, irrespective of menopausal status or other inclusion criteria. We report beta coefficients, which represent the average change in age (in years) of menopause for each chemical between menopausal women with EDC level ≥ 90^th^ percentile and those with EDC levels <90^th^ percentile.

**Dose-response analyses**. The distribution of levels of individual EDCs was highly variable; for example, levels of PCB-99 ranged from 0.65 ng/g to 304 ng/g lipid weight (Table 2). Thus, we took two approaches to assessing dose-response relationships between levels of EDC and outcome.[17] The first method examined the association of age of menopause with log-transformed concentrations of EDC. The second examined the association of age of menopause with EDC level categorized in deciles. For these analyses, the beta represents the change in mean age of menopause attributed to a one-unit change in the log concentrations of EDC or a one-decile increase in EDC, respectively (Table 3).

**Table 3:** Association between exposure to pervasive EDCs and earlier age of menopause in threshold and dose-response analyses.

EDC	Compound Name	N*	Threshold analysis		Dose-response analysis			
			EDC > 90th percentile†		Log EDC‡		EDC decile§	
			Average change in age of menopause beta (SE) in years	*P*	Average change in age of menopause beta (SE) in years	*P*	Average change in age of menopause beta (SE) in years	*P*
PCBs	PCB-74	268	−1.98 (1.00)	0.058	**−1.47 (0.603)**	**0.021**	−0.28 (0.214)	0.201
	PCB-99	248	**−3.08 (0.915)**	**0.002**	**−1.51 (0.504)**	**0.006**	−0.37 (0.180)	0.051
	PCB-105	249	**−1.86 (0.677)**	**0.010**	**−1.46 (0.467)**	**0.004**	**−0.36 (0.126)**	**0.008**
	PCB-118	249	**−2.46 (0.826)**	**0.006**	**−1.02 (0.446)**	**0.029**	−0.37 (0.189)	0.062
	PCB-138	250	**−2.21 (0.764)**	**0.007**	**−1.58 (0.429)**	**0.001**	**−0.71 (0.186)**	**<0.001**
	PCB-153	250	**−1.89 (0.799)**	**0.025**	**−1.40 (0.473)**	**0.006**	**−0.61 (0.200)**	**0.005**
	PCB-156	268	−0.52 (0.863)	0.553	**−1.92 (0.617)**	**0.004**	**−0.52 (0.169)**	**0.004**
	PCB-170	268	−0.85 (0.962)	0.385	**−1.21 (0.574)**	**0.045**	−0.26 (0.231)	0.272
	PCB-183	249	**−2.29 (0.817)**	**0.009**	**−1.58 (0.618)**	**0.016**	**−0.31 (0.145)**	**0.040**
Pesticides	p,p’-DDE	268	−1.42 (0.926)	0.136	**−0.67 (0.320)**	**0.046**	**−0.34 (0.162)**	**0.043**
	Beta-hexachloro-cyclohexane	177	**−1.90 (1.03)**	**0.075**	**−0.70 (0.138)**	**<0.001**	**−0.32 (0.078)**	**0.004**
	Mirex	177	−1.39 (0.823)	0.103	**−0.54 (0.084)**	**<0.001**	**−0.12 (0.049)**	**0.021**
Dioxin/furan	1,2,3,4,6,7,8-hepta-chlorodibenzofuran	212	**−1.77 (0.697)**	**0.024**	−0.72 (0.567)	0.178	−0.22 (0.193)	0.114
Phthalate	Mono-(2-ethyl-5-hydroxyhexyl)phthalate	153	**−3.80 (0)**	**<0.001**	**−0.35 (0)**	**<0.001**	**−0.17 (0)**	**<0.001**
	Mono-(2-ethyl-5-oxohexyl)phthalate	153	**−3.17 (0)**	**<0.001**	**−0.28 (0)**	**<0.001**	**−0.22 (0)**	**<0.001**

All estimates adjusted for age, race/ethnicity, body mass index, and current smoking status. Urine-based tests were also adjusted for urinary creatinine.

* Unweighted sample size.

† EDC exposure was defined as a binary variable > 90^th^ percentile.

‡ EDC value was log transformed and analyzed as a continuous variable.

§ EDC exposure was subdivided into deciles and analyzed as a continuous variable.

Bolded values indicate *P*-values <0.05.

### Secondary analysis

Because EDC levels in many women were measured years after the onset of menopause, we performed secondary analyses for the 15 chemicals with long half-lives or known reproductive toxicants identified in the primary analyses as being associated with an earlier age at menopause. This allowed us to shorten the length of time between exposure and outcome. We included all women age 45–55 years and used the same exclusion criteria listed above. The outcome of interest was menopausal status (dichotomized into menopausal or not menopausal). We computed the odds ratio of being menopausal attributable to each unit increase in log transformed EDC levels (Table 4).

**Table 4:** Secondary analysis for women 45–55 years of age with odds ratio of being menopausal as exposed to increasing logarithmic transformed EDC.

EDC	Compound Name	N	Odds Ratio	95% Confidence Interval
PCBs	PCB-74	222	**2.56**	**(1.54, 4.26)**
	PCB-99	222	**2.01**	**(1.26, 3.22)**
	PCB-105	222	**6.31**	**(2.68, 14.8)**
	PCB-118	222	**2.00**	**(1.30, 3.10)**
	PCB-138	222	**2.07**	**(1.24, 3.46)**
	PCB-153	222	**2.73**	**(1.60, 4.66)**
	PCB-156	222	**1.28**	**(1.14, 1.43)**
	PCB-170	222	**4.29**	**(2.22, 8.31)**
	PCB-183	222	**6.59**	**(2.31, 18.9)**
Pesticides	p,p’-DDE	225	1.44	(0.98, 2.13)
	Beta-hexachlorocyclohexane	225	1.43	(0.86, 2.38)
	Mirex	225	**3.00**	**(1.57, 5.73)**
Dioxin/furan	1,2,3,4,6,7,8-heptachlorodibenzofuran	225	1.06	(0.50, 2.2)
Phthalate	Mono-(2-ethyl-5-hydroxyhexyl)phthalate	550	1.00	(0.99, 1.00)
	Mono-(2-ethyl-5-oxohexyl)phthalate	550	0.810	(0.620, 1.06)

All estimates adjusted for age, race/ethnicity, body mass index, and current smoking status. Urine-based tests also adjusted for urinary creatinine. Bolded values represent significant odds ratio.

### Statistical analyses

All primary and secondary analyses were adjusted for age at the time of interview, race/ethnicity, body mass index, and current smoking status. Given the potential for a relationship between socioeconomic status and EDC exposure, we also evaluated poverty as a potential confounder. However, because poverty was not associated with a 10% change in any of the estimates of effect of individual EDCs (data not shown), socioeconomic status was not included in adjusted analyses. Given the use of multiple cycles of NHANES data, we computed combined weights in accordance with the NHANES Analytic Reporting Guidelines.[9] SAS (version 9.3, SAS Institute, Cary, NC) was used for statistical analyses.

## Results

The demographic characteristics of the women included in this study are presented in Table 1. The three subgroups of women (those who were menopausal; those included in the primary EDC analysis; and those included in the secondary analysis) were comparable in terms of mean age at menopause, socio-demographic and reproductive characteristics, and general health and behaviors to all women at least 30 years of age enrolled in NHANES from 1999–2008.

We analyzed the data reported for 111 EDCs among menopausal women, and focused this study on 35 EDCs with long half-lives and phthalates, known reproductive toxicants. Table 2 shows the 15 chemicals for which we found significant associations in any of the threshold or dose-response analyses. Overall, women with the highest EDC levels had ages of menopause 1.9 to 3.8 years earlier than those without. Nine of the 10 EDCs associated with a significantly earlier age at menopause in the threshold analysis were also statistically significant in one of the two dose-response analyses (Table 3). This result suggests that increasing serum- or urine-levels of EDCs, not just high absolute levels, are associated with loss of ovarian function.

In the secondary analysis of all women aged 45 to 55 years (Table 4), 11 out of 15 EDCs identified in our primary analyses were associated with significantly higher odds of being menopausal. Two had ORs indicative of increased likelihood of menopause but were not statistically significant, and two were not associated with likelihood of menopause.

## Discussion

Our study demonstrates a clinically significant association between levels of persistent EDCs and age at menopause in a large cross-sectional representative sample of US women. Nine PCBs, three pesticides, a furan, and two phthalates were significantly associated with earlier ages of menopause in at least one type of analysis. Fourteen of the 15 EDCs examined showed evidence of a dose-response relationship in at least one analysis, suggesting that increasing levels of environmental exposures of these chemicals could affect ovarian function. The observed magnitudes of effect of these 15 chemicals, ranging from 1.9 to 3.8 years earlier menopause, are larger than those previously documented for primary exposure to tobacco smoke, which has been shown in prior NHANES studies to associate with 0.8 to 1.4 years earlier menopause. [18,19] The odds of being menopausal among the restricted cohort of women (ages 45–55 years) ranged from 1.3 to greater than six fold for each one unit increase in the log EDC level. Although these chemicals may not be currently in production, there is likely still an ongoing exposure to many of these pervasive chemicals from our environment with an associated threat to human reproductive health.

EDCs may adversely affect ovarian function in several ways. One possibility is that EDCs slowly damage the follicular pool, leading to earlier menopause, similar to previously published studies on primary ovarian insufficiency.[20] An earlier result of such a process may be reduced fertility. For example, a recent prospective cohort study of 501 couples reported that exposures to several of the same PCBs that we identified (PCBs 118, 138, and 153) were associated with an approximately 20% adjusted reduction in fecundability.[14] Alternatively, EDCs may cause failure to establish a maximal oocyte pool in the case of exposure *in utero*, or they may increase follicular recruitment, leading to premature depletion of the follicular pools as is seen in some primary ovarian insufficiency models.[20,21] In support of this idea, adolescents who resided in an area of substantial industrial development and PCB exposure underwent earlier menarche and thelarche than their unexposed peers.[22] Additionally, mice exposed to diesel exhaust have a significant reduction in the proportion of primordial follicles, resulting in diminished ovarian reserve and an increased risk of premature menopause.[23] Further work with animal models is needed to better understand the mechanisms by which each of these chemicals disrupts endocrine function.

Our study identified 15 chemicals of potential interest, including nine PCBs. As a group, PCBs seem to cause the most concern for untoward reproductive consequences, though the mechanisms of action remain elusive. No EDC is thought to be a pure hormone agonist or antagonist, some PCBs have weak estrogenic activity,[24,25] and heavily chlorinated PCBs are thought to interact with the aryl hydrocarbon receptor.[26] Several studies have shown that exposure to PCBs reduces oocyte maturation, fertilization, and blastocyst development.[27–31] Additionally, the adverse reproductive effects of PCBs can be transmitted to subsequent generations in mice.[31] Increasing data suggest that each chemical has different mechanisms of action in varying tissues/organs, which complicates epidemiologic studies on EDC exposure. Finally, timing and duration of exposure is difficult to determine, and the effects of PCBs, many of which were banned from production in the 1970s,[32,33] may be long lasting given their pervasive nature throughout the environment.

The greatest strength of our study is the population of women studied. NHANES is a complex, stratified survey; sample weights, sampling strata, and sampling units were considered in all analyses to adjust for study design and create estimates that are representative of the US population. Furthermore, our analyses adjusted for age at the time of specimen collection, body mass index, race/ethnicity, and current smoking status. An additional strength of our study is our three-pronged analytical approach. Because there are no established safe thresholds for EDCs and little is known about their potential reproductive effects, we first examined the highest levels of EDC exposure. Second, we examined dose-response relationships in two ways: 1) as a continuous log-transformed variable; and 2) as a continuous variable based on decile of EDC level. Third, we performed a secondary analysis on the 15 identified chemicals of interest in a younger cohort of women to confirm and further strengthen our findings.

Our study has several limitations. First, our outcome was self-reported age at last menstrual period. However, although the possibility of differential recall exists, several studies have shown such data to be reliable.[16,32] Furthermore, all analyses adjusted for age at screening. Second, there was considerable delay between when menopause occurred and when EDC levels were measured. We attempted to reduce this limitation in our secondary analysis of women age 45–55 years; the women were in closer proximity to the natural age of menopause in the US (median 51.3)[34]. Furthermore, most of the chemicals that showed a significant association with menopause had long half-lives in humans (on the order of 10 years or more). Third, EDC data were not available for all eligible menopausal women, and NHANES laboratory cohorts were split into approximately three representative sets, each of which was evaluated for only a few classes of EDCs. This approach is cost-efficient but prevented combination of datasets across survey cycles, limiting our ability to evaluate cumulative effects of categories of EDCs and potentially reducing the power to detect other significant associations. Fourth, the NHANES data are cross-sectional; therefore, it is impossible to establish the temporal relationships necessary to establish causality. EDC levels were measured after the occurrence of menopause–no estimates from the relevant etiologic period before menopause are available. However, this study focused on chemicals with long half-lives, and prior studies have documented that a single measure provides considerable information on relative concentrations at distant times.[35] [36] Finally, despite performing multiple analyses, we did not adjust our alpha for multiple comparisons. We made this choice because our objective was to explore any potential association and report hypothesis-generating findings for future exploration of these harmful chemicals. Data regarding the pervasiveness of the chemicals highlighted in this study are limited by the innate difficulties in determining the half-lives of chemicals in different states and climates [37]. An important limitation of studies examining associations between single EDCs and diseases is that humans are exposed to multiple EDCs at any given time; these mixtures likely have a greater effect than any single EDC alone. Given that the cumulative effects and potential for synergism are unknown, we limited our analyses to individual chemicals. Similarly, dose-response relationships have not been established for the majority of the chemicals analyzed; future research should consider that selected chemicals might not exhibit traditional linear dose response curves. Additionally our findings are limited to the analysis available in the NHANES dataset. The associations noted in our analysis may also be influenced by a complex mixture of contaminants that have not yet been identified or measured in the NHANES database.

Our data demonstrate associations between 15 specific EDCs and earlier age at menopause–with menopause occurring as much as 3.8 years earlier in women with the highest EDC levels. Although many of these chemicals have been banned from production in the US because of their association with adverse health outcomes, some chemicals and other related chemicals are still produced globally and are pervasive and persistent. Although menopause timing is only one negative health impact that might be associated with EDC exposure, it is a very important one. Any early decline in ovarian function could increase rates of infertility and lead to earlier development of cardiovascular disease, osteoporosis, and other medical problems among women[38,39]. We need further critical evaluation of this topic in the form of animal models and prospective studies. Additional analyses examining the additive effects of genetic predispositions and environmental effects on reproductive health, as well as the potential for synergism between chemicals, are also important. We support the use of an approach that captures lifestyle, behavior, and macro-level exposures from conception onward to elucidate the impact of EDCs on human reproduction.[14,40] The health of future generations is at risk, and without further research in this area, those born today could be affected in decades to come.

## Supporting Information

S1 Table List of EDCs included in analysis, relative half-lives, and average change in age of menopause in threshold analyses for each chemical. (DOCX)

S2 Table List of EDCs excluded from analysis. (DOCX)
