# Supplementary material for: Persistent Organic Pollutants and Early Menopause in U.S. Women
Source: PLoS One. 2015 Jan 28;10(1):e0116057. doi: 10.1371/journal.pone.0116057 (PMC4309567; doi:10.1371/journal.pone.0116057)
Supplement: S1 Table — (DOCX) [file pone.0116057.s001.docx]

Supplementary Table 1 (S1). List of EDCs included in analysis, relative half-lives, and average change in age of menopause in threshold analyses for each chemical.

| **Category** | **NHANES code** | **Compound name** | **Biological Half-Life *** | |  |  |  |  |
| --- | --- | --- | --- | --- | --- | --- | --- | --- |
|  |  |  |  |  |  |  |  |  |
|  |  |  | **>1 year** | **Specimen** |  |  | **Log** |  |
|  |  |  |  | **Type** | **Beta †** | **P- value** | **Beta** | **P-value** |
| Dioxin | f03 | 2,3,4,7,8-pentachlorodibenzofuran | >1 year | Serum | -0.931 | 0.359 | -0.649 | 0.229 |
| Dioxin | f04 | 1,2,3,4,7,8-hexachlorodibenzofuran | >1 year | Serum | -1.29 | 0.177 | -0.694 | 0.346 |
| Dioxin | f08 | 1,2,3,4,6,7,8-heptachlorodibenzofuran | >1 year | Serum | **-1.77** | **0.0243** | -0.719 | 0.178 |
| Dioxin | tcd | 2,3,7,8- (tcdd) | >1 year | Serum | -0.115 | 0.947 | -0.127 | 0.884 |
| PCB | t028 | PCB-28 | >1 year | Serum | -2.11 | 0.163 | **-1.76** | **0.0433** |
| PCB | t052 | PCB-52 | >1 year | Serum | 0.445 | 0.742 | -0.0802 | 0.907 |
| PCB | t066 | PCB-66 | >1 year | Serum | -0.612 | 0.491 | -0.701 | 0.235 |
| PCB | t074 | PCB-74 | >1 year | Serum | -1.98 | 0.0577 | **-1.47** | **0.0208** |
| PCB | t099 | PCB-99 | >1 year | Serum | **-3.08** | **0.0021** | **-1.51** | **0.0057** |
| PCB | t101 | PCB-101 | >1 year | Serum | -0.371 | 0.805 | -0.121 | 0.861 |
| PCB | t105 | PCB-105 | >1 year | Serum | **-1.86** | **0.0103** | **-1.46** | **0.0041** |
| PCB | t118 | PCB-118 | >1 year | Serum | **-2.46** | **0.0058** | **-1.03** | **0.0288** |
| PCB | t128 | PCB-128 | >1 year | Serum | -1.42 | 0.295 | -1.03 | 0.319 |
| PCB | t138 | PCB-138 | >1 year | Serum | **-2.21** | **0.0072** | **-1.58** | **0.001** |
| PCB | t153 | PCB-153 | >1 year | Serum | **-1.89** | **0.0247** | **-1.39** | **0.0062** |
| PCB | t156 | PCB-156 | >1 year | Serum | -0.518 | 0.553 | **-1.92** | **0.0041** |
| PCB | t170 | PCB-170 | >1 year | Serum | -0.849 | 0.385 | **-1.21** | **0.0446** |
| PCB | t180 | PCB-180 | >1 year | Serum | -0.341 | 0.741 | -0.745 | 0.106 |
| PCB | t183 | PCB-183 | >1 year | Serum | **-2.29** | **0.0091** | **-1.58** | **0.0161** |
| Pesticides | hcb | Hexachlorobenzene | >1 year | Serum | -0.0083 | 0.993 | 0.101 | 0.853 |
| Pesticides | hpe | Heptachlor epoxide | >1 year | Serum | 0.344 | 0.584 | -0.291 | 0.547 |
| Pesticides | pde | p,p'-DDE | >1 year | Serum | -1.42 | 0.136 | **-0.667** | **0.0464** |
| Pesticides | pdt | p,p'-DDT | >1 year | Serum | -0.977 | -0.402 | -0.673 | 0.161 |
| Pesticides | bhc | Beta-hexachlorocyclohexane | >1 year | Serum | -1.904 | 0.0754 | **-0.702** | **<0.001** |
| Pesticides | mir | Mirex | >1 year | Serum | -1.39 | 0.103 | **-0.542** | **<0.001** |
| Pesticides | odt | o,p’-DDT | >1 year | Serum | **-3.18** | **0.0115** | **-1.82** | **<0.001** |
| Pesticides | die | Dieldrin | >1 year | Serum | 1.43 | 0.222 | -0.571 | 0.384 |
| Pesticides | end | Endrin | >1 year | Serum | -1.25 | 0.556 | -2.59 | 0.0934 |
|  |  |  |  |  |  |  |  |  |
| **Category** | **NHANES code** | **Compound name** | **Biological Half-Life *** | **Specimen** | **Beta †** | **P-value** | **Log** | **P-value** |
|  |  |  | **<1 year** | **Type** |  |  | **Beta** |  |
| Dioxin | f01 | 2,3,7,8-tetrachlorodibenzofuran | <1 year | Serum | -1.57 | 0.489 | 0.0793 | 0.915 |
| Dioxin | f02 | 1,2,3,7,8-pentachlorodibenzofuran | <1 year | Serum | -0.629 | 0.651 | -0.464 | 0.583 |
| Phytoestrogen | dma | O-Desmethylangolensin | <1 year | Urine | -1.46 | 0.426 | -0.137 | 0.481 |
| Phytoestrogen | equ | Equol | <1 year | Urine | 1.24 | 0.177 | 0.177 | 0.521 |
| Phytoestrogen | etd | Enterodiol | <1 year | Urine | N/A | N/A | 0.0076 | 0.976 |
| Phytoestrogen | etl | Enterolactone | <1 year | Urine | -0.156 | 0.911 | 0.0657 | 0.752 |
| Phytoestrogen | gns | Genistein | <1 year | Urine | 0.7204 | 0.392 | 0.137 | 0.574 |
| Phytoestrogen | daz | Daidzein | <1 year | Urine | -1.19 | 0.385 | -0.0248 | 0.924 |
| Phenols | t24d | 2, 4-Dichlorophenoxyacetic acid | <1 year | Urine | -0.472 | 0.733 | -0.353 | 0.295 |
| Phenols | par | Paranitrophenol | <1 year | Urine | 1.39 | 0.0818 | 0.396 | 0.0864 |
| Phenols | dee | DEET | <1 year | Urine | 2.65 | 0.0089 | 0.327 | 0.304 |
| Phenols | t4fp | 4-fluoro-3-phenoxybenzoic | <1 year | Urine | -0.604 | 0.691 | -0.319 | 0.679 |
| Pesticides | tna | Trans-nonachlor | <1 year | Serum | -0.511 | 0.574 | -0.522 | 0.336 |
| Pesticides | op1 | Dimethylphosphate | <1 year | Urine | 1.29 | 0.324 | 0.412 | 0.176 |
| Pesticides | op2 | Diethylphosphate | <1 year | Urine | 2.77 | 0.0024 | 0.376 | 0.251 |
| Pesticides | op4 | Diethylthiophosphate | <1 year | Urine | -4.005 | 0.0068 | -0.124 | 0.739 |
| Pesticides | ala | Alachor mercapturate | <1 year | Urine | 2.49 | 0.0005 | -0.294 | 0.147 |
| Pesticides | atz | Atrazine mercapturate | <1 year | Urine | -1.63 | 0.492 | -0.599 | 0.0149 |
| Pesticides | mal | Malathion diacid | <1 year | Urine | 0.943 | 0.204 | -0.222 | 0.348 |
| Pesticides | cpm | 3,5,6-trichloropyridinol | <1 year | Urine | -0.919 | 0.609 | 0.606 | 0.116 |
| Pesticides | diz | Oxypyrimidine | <1 year | Urine | 0.571 | 0.731 | 0.0663 | 0.411 |
| Pesticides | ghc | Gamma-hexachloro-cyclohexane | <1 year | Serum | -0.0445 | 0.982 | -0.146 | 0.902 |
| Pesticides | oxy | Oxychlordane | <1 year | Serum | -0.561 | 0.523 | -0.843 | <0.001 |
| Pesticides | ald | Aldrin | <1 year | Serum | -3.02 | 0.0911 | -2.99 | 0.0787 |
| PAH | p01 | 1-hydroxynapthalene | <1 year | Urine | -1.03 | <0.001 | 0.0902 | <0.001 |
| PAH | p05 | 3-hydroxyphenanthrene | <1 year | Urine | -1.298 | <0.001 | -0.991 | <0.001 |
| PAH | p10 | 1-hydroxypyrene | <1 year | Urine | -3.65 | <0.001 | -0.948 | <0.001 |
| Phthalate | mhh | Mono-(2-ethyl-5-hydroxyhexyl)phthalate | <1 year | Urine | **-3.799** | **<0.001** | **-0.345** | **<0.001** |
| Phthalate | moh | Mono-(2-ethyl-5-oxohexyl)phthalate | <1 year | Urine | **-3.17** | **<0.001** | **-0.279** | **<0.001** |
| Phthalate | mib | Mono-isobutyl phthalate | <1 year | Urine | **6.78** | **<0.001** | **0.469** | **<0.001** |
| Phthalate | mbp | Mono-n-butyl phthalate | <1 year | Urine | -0.659 | 0.694 | -0.359 | 0.297 |
| Phthalate | mep | Mono-ethyl phthalate | <1 year | Urine | -0.185 | 0.887 | -0.0012 | 0.996 |
| Phthalate | mhp | Mono-(2-ethyl)-hexyl phthalate | <1 year | Urine | 0.539 | 0.777 | 0.164 | 0.683 |
| Phthalate | mop | Mono-n-octyl phthalate | <1 year | Urine | -1.85 | 0.319 | -1.32 | 0.287 |
|  |  |  |  |  |  |  |  |  |
| **Category** | **NHANES code** | **Compound name** | **Biological Half-Life *** | **Specimen** | **Beta †** | **P-value** | **Log** | **P-value** |
|  |  |  | **Unknown**  **Half-Life** | **Type** |  |  | **Beta** |  |
| Dioxin | d01 | 1,2,3,7,8-pentachlorodibenzo-p-dioxin | N/A | Serum | -1.16 | 0.0714 | -1.003 | 0.0114 |
| Dioxin | d03 | 1,2,3,6,7,8-hexachlorodibenzo-p-dioxin | N/A | Serum | -1.45 | 0.143 | -0.3603 | 0.396 |
| Dioxin | d04 | 1,2,3,7,8,9-hexachlorodibenzo-p-dioxin | N/A | Serum | -0.525 | 0.579 | -0.488 | 0.304 |
| Dioxin | d05 | 1,2,3,4,6,7,8-heptachlorodibenzo-p-dioxin | N/A | Serum | -0.444 | 0.6704 | -0.0275 | 0.956 |
| Dioxin | d07 | 1,2,3,4,6,7,8,9-octachlorodibenzo-p-dioxin | N/A | Serum | -0.778 | 0.273 | -0.557 | 0.335 |
| Dioxin | f06 | 1,2,3,7,8,9-hexachlorodibenzofuran | N/A | Serum | -0.706 | 0.671 | -0.289 | 0.718 |
| Dioxin | f07 | 2,3,4,6,7,8-hexachlorodibenzofuran | N/A | Serum | -0.769 | 0.428 | -0.246 | 0.719 |
| Dioxin | f10 | 1,2,3,4,6,7,8,9-octachlorodibenzofuran | N/A | Serum | -0.292 | 0.867 | -0.736 | 0.477 |
| Dioxin | hxc | 3,3',4,4',5,5' (hxcb) | N/A | Serum | 0.8699 | 0.296 | 0.201 | 0.754 |
| Dioxin | d02 | 1,2,3,6,7,8-(hxcdd) | N/A | Serum | -0.579 | 0.463 | 0.0278 | <0.001 |
| Dioxin | f09 | 1,2,3,4,7,8,9-(hpcdf) | N/A | Serum | -0.292 | 0.765 | 1.98 | <0.001 |
| PCB | pcb | 3,3',4,4',5-pentachlorobiphenyl | N/A | Serum | -1.595 | 0.044 | -0.124 | 0.763 |
| PCB | tc2 | 3,4,4',5-tetrachlorobiphenyl | N/A | Serum | -1.02 | 0.563 | -0.313 | 0.693 |
| PCB | t146 | PCB-146 | N/A | Serum | -1.72 | 0.0496 | -1.57 | 0.0081 |
| PCB | t157 | PCB-157 | N/A | Serum | -2.88 | 0.0021 | -1.94 | 0.0145 |
| PCB | t167 | PCB-167 | N/A | Serum | -1.53 | 0.236 | -1.46 | 0.0428 |
| PCB | t172 | PCB-172 | N/A | Serum | -3.3 | 0.0135 | -2.34 | 0.0052 |
| PCB | t177 | PCB-177 | N/A | Serum | -1.98 | 0.0601 | -1.37 | 0.0412 |
| PCB | t178 | PCB-178 | N/A | Serum | -2.14 | 0.0532 | -1.19 | 0.167 |
| PCB | t187 | PCB-187 | N/A | Serum | -1.13 | 0.232 | -1.12 | 0.0269 |
| PCB | t087 | PCB-87 | N/A | Serum | -3.18 | 0.0545 | -2.55 | 0.144 |
| PCB | t110 | PCB-110 | N/A | Serum | -1.67 | 0.313 | -1.57 | 0.303 |
| PCB | t149 | PCB-149 | N/A | Serum | -1.67 | 0.314 | -1.87 | 0.237 |
| PCB | t151 | PCB-151 | N/A | Serum | -3.05 | 0.0605 | -2.55 | 0.144 |
| PCB | t189 | PCB-189 | N/A | Serum | -3.05 | 0.0605 | -2.51 | 0.162 |
| PCB | t194 | PCB-194 | N/A | Serum | -1.92 | 0.0893 | -0.777 | 0.436 |
| PCB | t195 | PCB-195 | N/A | Serum | -1.08 | 0.536 | -2.23 | 0.185 |
| PCB | t196 | PCB-196 | N/A | Serum | -1.22 | 0.198 | -1.05 | 0.164 |
| PCB | t199 | PCB-199 | N/A | Serum | -2.15 | 0.111 | -1.005 | 0.238 |
| PCB | t206 | PCB-206 | N/A | Serum | -0.207 | 0.873 | -1.24 | 0.349 |
| Phthalate | mnm | Mono-n-methyl phthalate | N/A | Urine | -3.99 | <0.001 | -0.503 | <0.001 |
| Phthalate | mc1 | Mono-(3-carboxypropyl) phthalate | N/A | Urine | -0.0377 | <0.001 | N/A | N/A |
| Phthalate | mcp | Mono-cyclohexyl phthalate | N/A | Urine | 8.23 | 0.0083 | -0.206 | 0.777 |
| Phthalate | mnp | Mono-isononyl phthalate | N/A | Urine | 0.879 | 0.563 | -0.0987 | 0.916 |
| Phthalate | mzp | Mono-benzyl phthalate | N/A | Urine | 0.414 | 0.779 | -0.172 | 0.582 |
| Phenols | t25t | 2,4,5-Trichlorophenoxyacetic acid | N/A | Urine | 1.09 | 0.399 | -1.11 | 0.0158 |
| Phenols | cbf | Carbofuranphenol | N/A | Urine | 3.63 | 0.0007 | -0.606 | 0.427 |
| Phenols | ppx | 2-isopropoxyphenol | N/A | Urine | N/A | N/A | 1.48 | <0.001 |
| Phenols | opm | 3-phenoxybenzoic acid | N/A | Urine | 1.96 | 0.0386 | 0.698 | 0.0092 |
| Pesticides | op3 | Dimethylthiophosphate | N/A | Urine | 1.35 | 0.268 | 0.141 | 0.375 |
| Pesticides | op5 | Dimethyldithiophosphate | N/A | Urine | 2.28 | 0.0026 | 0.295 | 0.0954 |
| Pesticides | op6 | Diethyldithiophosphate | N/A | Urine | -0.117 | 0.901 | 0.0575 | 0.856 |
| Pesticides | ccc | cis dichlorovnl-dimeth carboacid | N/A | Urine | 2.35 | 0.0048 | 0.785 | 0.0248 |
| Pesticides | tcc | trans dichlorovnl-dimeth carboacid | N/A | Urine | 2.89 | 0.0001 | 0.879 | 0.0245 |
| PAH | p02 | 2-hydroxynapthalene | N/A | Urine | -1.296 | <0.001 | -0.135 | <0.001 |
| PAH | p03 | 3-hydroxyfluorene | N/A | Urine | 1.51 | <0.001 | -0.694 | <0.001 |
| PAH | p04 | 2-hydroxyfluorene | N/A | Urine | 0.0427 | <0.001 | -0.981 | <0.001 |
| PAH | p06 | 1-hydroxyphenanthrene | N/A | Urine | 0.5096 | <0.001 | -1.11 | <0.001 |
| PAH | p07 | 2-hydroxyphenanthrene | N/A | Urine | -0.236 | <0.001 | N/A | N/A |

All estimates adjusted for age, race/ethnicity, body mass index, and current smoking status. Urine-based tests were also adjusted for urinary creatinine.

* Half-life determined based on available literature from the Agency for Toxic Substances & Disease Registry (CDC)

**†** EDC exposure was defined as a binary variable > 90^th^ percentile
