# Supplementary material for: Persistent Organic Pollutants and Early Menopause in U.S. Women
Source: PLoS One. 2015 Jan 28;10(1):e0116057. doi: 10.1371/journal.pone.0116057 (PMC4309567; doi:10.1371/journal.pone.0116057)
Supplement: S2 Table — (DOCX) [file pone.0116057.s002.docx]

Supplementary Table 2 (S2). List of EDCs excluded from analysis.

| **Category** | **NHANES code** | **Excluded Compound name** | **Biological Half-Life *** | **Specimen**  **Type** |
| --- | --- | --- | --- | --- |
| Phthalate | cop | Mono(carboxyoctyl) phthalate | <1 year | Urine |
| Phthalate | cnp | Mono(carboxynonyl) phthalate | <1 year | Urine |
| Flame retardant/surfactant | pfoa | Perfluorooctanoic acid | >1 year | Serum |
| Flame retardant/surfactant | pfos | Perfluorooctane sulfonic acid | >1 year | Serum |
| Phenols | pcp | Pentachlorophenol | <1 year | Urine |
| Phenols | dcb | 2,4-dichlorophenol | <1 year | Urine |
| Phenols | t3tb | 2,4,6-trichlorophenol | <1 year | Urine |
| Phenols | bph | Bisphenol A | <1 year | Urine |
| Phenols | bp3 | Benzophenone-3 | <1 year | Urine |
| Phenols | trs | Triclosan | <1 year | Urine |
| Flame retardant | br4la | 2,2',3,4,4'-pentabromodiphenyl ether | <1 year | Serum |
| Flame retardant | br5la | 2,2',4,4',5-pentabromodiphenyl ether | <1 year | Serum |
| Flame retardant | br6la | 2,2',4,4',6-pentabromodiphenyl ether | <1 year | Serum |
| Flame retardant | br8la | 2,2',4,4',5,6'-hexabromodiphenyl ether | <1 year | Serum |
| Flame retardant | br9la | 2,2',3,4,4',5',6-heptabromodiphenyl ether | <1 year | Serum |
| Flame retardant | br66l | 2,3',4,4'-tetrabromodiphenyl ether | <1 year | Serum |
| Phthalate | ecp | Mono-2-ethyl-5-carboxypentyl phthalate | N/A | Urine |
| Phenols | t14d | 2,5-dichlorophenol | N/A | Urine |
| Phenols | opp | O-Phenyl phenol | N/A | Urine |
| Phenols | t1tb | 2,4,5-trichlorophenol | N/A | Urine |
| Phenols | 4to | 4-tert-octylphenol | N/A | Urine |
| Phenols | bup | Butyl paraben | N/A | Urine |
| Phenols | epb | Ethyl paraben | N/A | Urine |
| Phenols | mp | Methyl paraben | N/A | Urine |
| Phenols | ppb | Propyl paraben | N/A | Urine |
| Pesticides | cb3 | dibromovinyl-dimeth prop carboacid | N/A | Urine |
| Pesticides | ape | Acephate | N/A | Urine |
| Pesticides | bsm | Bensulfuron methyl | N/A | Urine |
| Pesticides | chs | Chlorsulfuron | N/A | Urine |
| Pesticides | emm | Ethametsulfuron methyl | N/A | Urine |
| Pesticides | etu | Ethylenethio Urea | N/A | Urine |
| Pesticides | frm | Foramsulfuron | N/A | Urine |
| Pesticides | hls | Halosulfuron | N/A | Urine |
| Pesticides | mmi | Methamidophos | N/A | Urine |
| Pesticides | msm | Mesosulfuron methyl | N/A | Urine |
| Pesticides | mtm | Metsulfuron methyl | N/A | Urine |
| Pesticides | mto | Dimethoate | N/A | Urine |
| Pesticides | nos | Nicosulfuron | N/A | Urine |
| Pesticides | omo | O-Methoate | N/A | Urine |
| Pesticides | oxs | Oxasulfuron | N/A | Urine |
| Pesticides | pim | Primisulfuron methyl | N/A | Urine |
| Pesticides | pro | Prosulfuron | N/A | Urine |
| Pesticides | ptu | Propylenethio Urea | N/A | Urine |
| Pesticides | rim | Rimsulfuron | N/A | Urine |
| Pesticides | smm | Sulfometuron methyl | N/A | Urine |
| Pesticides | ssf | Sulfosulfuron | N/A | Urine |
| Pesticides | thf | Thifensulfuron methyl | N/A | Urine |
| Pesticides | tra | Triasulfuron | N/A | Urine |
| Pesticides | trn | Tribenuron methyl | N/A | Urine |
| PAH | p17 | 9-hydroxyfluorene | N/A | Urine |
| PAH | p19 | 4-hydroxyphenanthrene | N/A | Urine |
| Flame retardant | bb1la | 2,2',4,4',5,5'-hexbrombiphenyl | N/A | Serum |
| Flame retardant | br1la | 2,2',4-tribromodiphenyl ether | N/A | Serum |
| Flame retardant | br2la | 2,4,4'-tribromodiphenyl ether | N/A | Serum |
| Flame retardant | br3la | 2,2',4,4'-tetrabromodiphenyl ether | N/A | Serum |
| Flame retardant | br7la | 2,2',4,4',5,5'-hexabromodiphenyl ether | N/A | Serum |
| Flame retardant/surfactant | pfhs | Perfluorohexane sulfonic acid | N/A | Serum |
| Flame retardant/surfactant | ePAH | 2-(N-Ethyl-perfluorooctane sulfonamido) acetic acid | N/A | Serum |
| Flame retardant/surfactant | mPAH | 2-(N-Methyl-perfluorooctane sulfonamido) acetic acid | N/A | Serum |
| Flame retardant/surfactant | pfde | Pefluorodecanoic acid | N/A | Serum |
| Flame retardant/surfactant | pfhp | Perfluoroheptanoic acid | N/A | Serum |
| Flame retardant/surfactant | pfna | Perfluorononanoic acid | N/A | Serum |
| Flame retardant/surfactant | pfsa | Perfluorooctane sulfonamide | N/A | Serum |
| Flame retardant/surfactant | pfua | Perfluoroundecanoic acid | N/A | Serum |
| Flame retardant/surfactant | pfdo | Perflurododecanoic acid | N/A | Serum |
